# A case of dysphagia

**DOI:** 10.11604/pamj.2014.17.56.3876

**Published:** 2014-01-25

**Authors:** George Sarin Zacharia, Sandesh Kolassery

**Affiliations:** 1 Department of Medical Gastroenterology, Government Medical College, Calicut, Kerala, India

**Keywords:** Dysphagia, esophageal strictures, radiation therapy

## Image in medicine

Esophageal strictures commonly complicate radiation therapy for neck and thoracic malignancies. We here report a case of radiation induced stricture esophagus and illustrate the classical barium esophagogram in this condition. 58 year old male with past history of carcinoma esophagus treated with radiotherapy had presented with recurrence of dysphagia. Dysphagia was insidious in onset and was predominantly for solids than liquids. Physical examination was normal except for gross emaciation. Hemogram and routine biochemical panel were within normal limits. Barium swallow revealed significant mid-esophageal luminal narrowing with significant contrast pooling above. Upper gastrointestinal endoscopy revealed luminal narrowing at 25cms from the incisors; it was not possible to negotiate through the stricture. At endoscopy mucosa appeared normal except for minimal erythema. Mucosal biopsies revealed only normal stratified squamous epithelium and no evidence of neoplasm. Contrast computed tomography revealed no evidence of tumor recurrence or metastasis or lymphadenopathy. Patient was managed with serial dilatation with Savary Guillard dilators. Patient improved with treatment and is currently able to take oral feeds. Stricture is commonly reported to develop in 2-3 weeks to 4-8 months after radiation exposure, though late presentations also can occur. In patients presenting with dysphagia following radiotherapy for carcinoma esophagus it is always important to rule out residual or recurrent neoplasm with endoscopy and imaging. Esophageal dilatation with or without stenting is the current treatment of choice.

**Figure 1:**
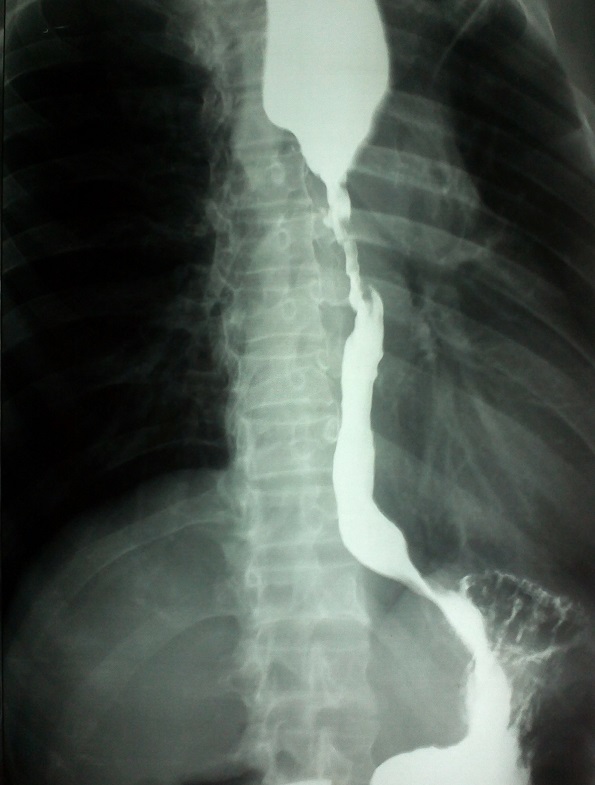
Barium esophagogram showing mid-esophageal stricture

